# An exploratory assessment model for preventing individual extreme violent crimes from a social control perspective—a qualitative study of four Chinese cases

**DOI:** 10.3389/fpsyg.2025.1593232

**Published:** 2025-12-02

**Authors:** Gu Anqi

**Affiliations:** Department of Investigation, Jiangsu Police Institute, Nanjing, Jiangsu, China

**Keywords:** individual extreme violent crime, formal control, informal control, self-control, prevention and control evaluation model

## Abstract

**Introduction:**

Individual extreme violent crimes pose a severe threat to public safety. From a social control perspective, this paper explores the dimensions of “formal control,” “informal control,” and “self-control”. Drawing on criminological paradigms, it proposes an exploratory prevention and control evaluation model for such crimes.

**Methods:**

The model’s utility is preliminarily tested through an in-depth analysis of four illustrative cases in China, exploring failures in institutional management, support ties, and self-regulation.

**Results:**

The analysis suggests that the suspects in these cases exhibited low self-regulation abilities and weak psychological adjustment when facing life challenges. These extreme crimes appeared prone to occur when informal social ties were weakened or broken, even while some forms of formal control were present.

**Discussion:**

By applying a social control framework, this study offers a new analytical tool that complements traditional, macro-level crime prevention research. Rather than providing definitive solutions, the model is intended to serve as a heuristic device for identifying systemic weaknesses, thereby promoting a more nuanced understanding of extreme violence both theoretically and empirically.

## 1 Introduction

Public safety is a crucial manifestation of the modernization of the social governance system and governance capacity, directly related to social stability and people’s well-being (Zhang et al., 2019). With the socio-economic development and continuous improvement of the comprehensive social security governance system, the number and incidence rate of traditional criminal offenses has been declining. However, individual extreme violent crime, as a special form of criminality, has exhibited a frequent occurrence trend globally in recent years. Although the number of such cases is relatively small, due to their suddenness, cruelty, and widespread social impact, they easily trigger public panic and media attention, posing a common challenge for public safety governance in various countries. From a global perspective, research on individual extreme violent crime primarily focuses on Lone Wolf Terrorism (LWT) (Simon, 2015) Hate Crime (Vergani et al., 2024), and Random Killing, exemplified by cases such as the “mass shootings” in the United States (Silver, 2024), “motiveless crimes” in South Korea (Eun-Young and Soon-Sil, 2019), and “indiscriminate killings” in Japan (Im Soo and Eung-Ryul, 2022), all of which reflect the diversity and complexity of individual extreme violent crime. These studies indicate that perpetrators often adopt extreme behaviors due to political beliefs, religious faith, racial hatred, psychological trauma, social exclusion, or life difficulties. However, their motives, goals, and means vary significantly across different cultures, institutions, and social backgrounds. Nevertheless, existing research primarily focuses on Western social contexts, lacking in-depth exploration of non-Western countries, especially the Chinese context.

In China, individual extreme violent crime typically refers to severe criminal acts committed by a single perpetrator for purposes such as venting personal grievances, retaliating against society, or creating an impact, employing extreme violent means such as explosion, stabbing, arson, shooting, and vehicular ramming against society or others. Unlike “lone wolf” terrorist attacks, individual extreme violent crime in China does not involve political demands. Compared with hate crimes, its motives are not based on discriminatory factors such as race, religion, or gender. The perpetrators of individual extreme violent crime in China are mostly males aged 30 to 50, commonly characterized by low income, low education, life difficulties, and paranoid personality traits. A minority of their targets are similar to those in indiscriminate homicide crimes, with the sole purpose of retaliating against society, and the attack targets are completely random, mostly being socially vulnerable groups such as the elderly, women, or children. Most cases, however, involve causing casualties to individuals closely related to or unrelated to the specific target during the attack, with the targets exhibiting both specificity and randomness. In addition to the various obstacles faced in the life courses of criminal suspects, there are deficiencies in the social prevention and control system (Martin et al., 2016). Existing research mostly explores the causes and prevention strategies of individual extreme violent crime from a macro perspective, focusing on the control mechanisms of police departments while neglecting informal control aspects such as family and community factors. Meanwhile, research on criminal suspects only focuses on their psychological conditions (Jordan and McNiel, 2021) or mental status (Edwards and Kotera, 2023), while their daily life experiences at the micro level have not been comprehensively analyzed and presented, leading to an inadequate analysis of criminal suspects’ difficulties and self-control abilities. The limitations of these studies restrict a comprehensive understanding of individual extreme violent crime and also affect the effectiveness of prevention and control strategies.

Therefore, this paper introduces the theoretical perspective of “social control” to explore the regulatory systems, support ties, and the real-life predicaments and self-prevention abilities of suspects in personal extreme violent crime cases, and builds a personal extreme violent crime prevention and control evaluation model based on this. In recent years, “social control theory” has received increasing academic attention in the field of criminology research. Social control theory was formally proposed by American sociologist Ross, who believed that the inadequacy of social control forces can lead to the occurrence of criminal behavior (Ross, 2017). Social control can be divided into external control (formal control and informal control) and internal control. The subject of formal control refers to organizations and personnel specializing in social control. Informal social control can be divided into attachment, commitment, engagement, and belief according to its mechanisms. Internal control refers to self-control (Benson, 2017). Through fieldwork research on typical cases, this study summarizes the relevant elements of formal control, informal control, and self-control. Using these as coordinate axes, a spatial rectangular coordinate system is established to form an evaluation model for the prevention and control effectiveness of individual extreme violent crimes. The theoretical perspective and analytical tool of social control can provide insights into the multidimensional difficulties and self-control barriers faced by criminal suspects, enabling a more comprehensive and profound understanding of the institutional and non-institutional factors contributing to individual loss of control. Additionally, it allows for an effective evaluation of the social prevention and control system.

Currently, individual extreme violent crimes in China have garnered significant attention from the government and national leaders. Following the “Zhuhai Vehicle Ramming Incident on November 12, 2024” in China, General Secretary Xi Jinping attached great importance to it and issued important instructions, stressing that all regions and relevant departments should deeply learn from the lesson, draw inferences about other cases from one instance, strengthen prevention and control at the source of risks, promptly resolve contradictions and disputes, and strictly prevent the occurrence of extreme cases. The report of the 20th National Congress of the Communist Party of China and the Decisions of the Third Plenary Session of the 20th Central Committee of the Communist Party of China both made strategic deployments for improving the public security governance mechanism, promoting the transformation of the public security governance model from passive response after the event to proactive prevention beforehand. In China, some scholars have conducted research on individual extreme violent crimes, covering multiple disciplines including informatics (Ma and Xu, 2024), criminology (Li et al., 2024; Xu, 2023), public security science (Deng and Kang, 2024), criminal justice (Wu, 2024), transportation science (Xu and Wang, 2020), and psychology (Zeng, 2024; Zhao and Kang, 2022). Most of these studies employed qualitative research methods, while a few utilized quantitative research. Given that China’s unique sociocultural dynamics may differ from those in the West in terms of their impact on personal extreme violent crimes, the scarcity of research on personal extreme violent crimes in China is concerning. Existing research still lacks a sufficient understanding of the prevention and control assessment of individual extreme violent crimes in China. On the one hand, in Chinese society, which advocates harmony and stability, discussions about homicide are often considered sensitive topics, which hinders the unfolding of related discussions (Chan et al., 2019). On the other hand, although the development of various disciplines has contributed to the early stages of criminology, the unclear interconnections among these fields indicate a lack of specialized scholars in Chinese criminology. Therefore, these factors collectively indicate that crime research in China faces significant challenges, especially in focusing more academic attention on more specific crime subtypes (Shuai and Liu, 2023). Thus, these factors collectively indicate that crime research in China faces significant challenges, particularly in focusing more academic attention on more specific crime subtypes. This also explains why research in the field of individual extreme violent crimes is so scarce in the Chinese context, highlighting the need for more academic exploration to develop targeted intervention and prevention strategies for China.

This study aims to construct an assessment model for the prevention and control of individual extreme violent crimes based on social control theory. Furthermore, the study evaluates the effectiveness of prevention and control measures for four individual extreme violent crime cases that occurred in China using this model, to identify existing issues in current prevention and control efforts. Firstly, what is the current state of formal prevention and control mechanisms for individual extreme crimes, and what deficiencies exist? Secondly, what is the state of the criminal suspects’ social ties, and are they weak? Thirdly, what difficulties do criminal suspects face in their daily lives, and what is their level of self-control? Answering these questions requires a deep understanding of criminal intent, which largely relies on qualitative verbal or textual narratives, rather than solely on quantitative data. To this end, this study conducted field visits across four provinces and obtained a large amount of primary and secondary data, including personal interviews, prosecution records, interrogation transcripts, victim testimonies, and witness statements. This study hopes to identify factors lacking in prevention and control, enhance academic understanding of individual extreme violent crimes, and provide qualitative insights to improve the theoretical framework aimed at preventing individual extreme violent crimes.

## 2 Theoretical framework and model construction

### 2.1 Social control theory

Using a theoretical framework to explain social phenomena has always been a strategy in social science research. Social control theory originates from “Social Control: An Inspection of the Cornerstone of Order” published by American sociologist Ross (1901). The definition of social control can be broadly or narrowly defined. In a broad sense, it refers to all human practices and arrangements that contribute to the formation and maintenance of social order, especially those that influence compliance (Hirschi, 2015). In a narrow sense, it is a category relative to social deviance and crime, referring to organized responses to deviant behavior, i.e., the process of regulating and restraining deviants (Black, 2010). Any unwelcome behavior is considered deviant, and crime is deviant behavior that also violates criminal law. Social control over crime is an important aspect of social control (Costello and Laub, 2020). The means of social control are diverse and can be classified into external control and internal control. External social control refers to the use of certain norms and corresponding means and methods by social institutions and organizations to constrain and guide the behavior and values of deviant members, ensuring that they maintain good social behavior under the guidance, constraint, and control of social norms. Based on the implementing entity, external social control can be further divided into formal control and informal control (Cho, 2024). Formal control refers to organizations and personnel specifically responsible for social control, such as police and courts, while informal control encompasses relationships formed within primary groups based on value norms such as culture, religion, and ethics. Internal control, also known as self-control, is the process of guiding individuals to self-motivate and act in a compliant manner (Akers, 1991).

Social control in the criminological sense, also known as “Social Bond Theory,” is one of the important theories of the criminological sociology school (Wiatrowski, 1978). It is a theory that explains why people do not commit crimes, with the representative figure being contemporary American criminologist Gottfredson and Hirschi (1990). Hirschi summarizes social control as “social bonds” and “self-control.” Social bonds consist of four components. First, attachment (emotion), which refers to an individual’s connection with the outside world, especially with family and friends. If an individual does not experience feelings of closeness, they will be less willing to be bound by common contracts, leading to a higher risk of committing crimes. Second, commitment (cost), refers to the time, economic, and human resources that an individual invests in daily activities such as attending school and working, to obtain wealth, knowledge, and social evaluation. The more such investments, the more effective the control over criminal intent. Third, involvement (job stability), in contrast to loneliness, Hirschi believes that a healthy social structure reduces the time, space, and energy available for individuals to commit crimes and that idleness is the fertile ground for crime. Fourth, belief (constraint of social norms), which refers to the recognition and inheritance of laws and regulations, social ethics, and other values, rather than religious beliefs. In “A General Theory of Crime,” Hirschi points out that crime is the product of suitable opportunities combined with low levels of self-control. This means that when an individual has the opportunity to commit a crime, they may not necessarily do so; they also need to overcome the minimum standard of personal self-control. Differences in the tendency to commit crimes may lie in differences in individuals’ levels of self-control. Hirschi summarizes the characteristics of individuals with low self-control ability into three points: simplistic thinking, impulsive emotions, and egocentric values. Social bonds and self-control have an intersecting relationship; social bonds are an important manifestation of self-control, while self-control is a deeper influencing factor of social bonds (Buker, 2011).

**Figure 1:**
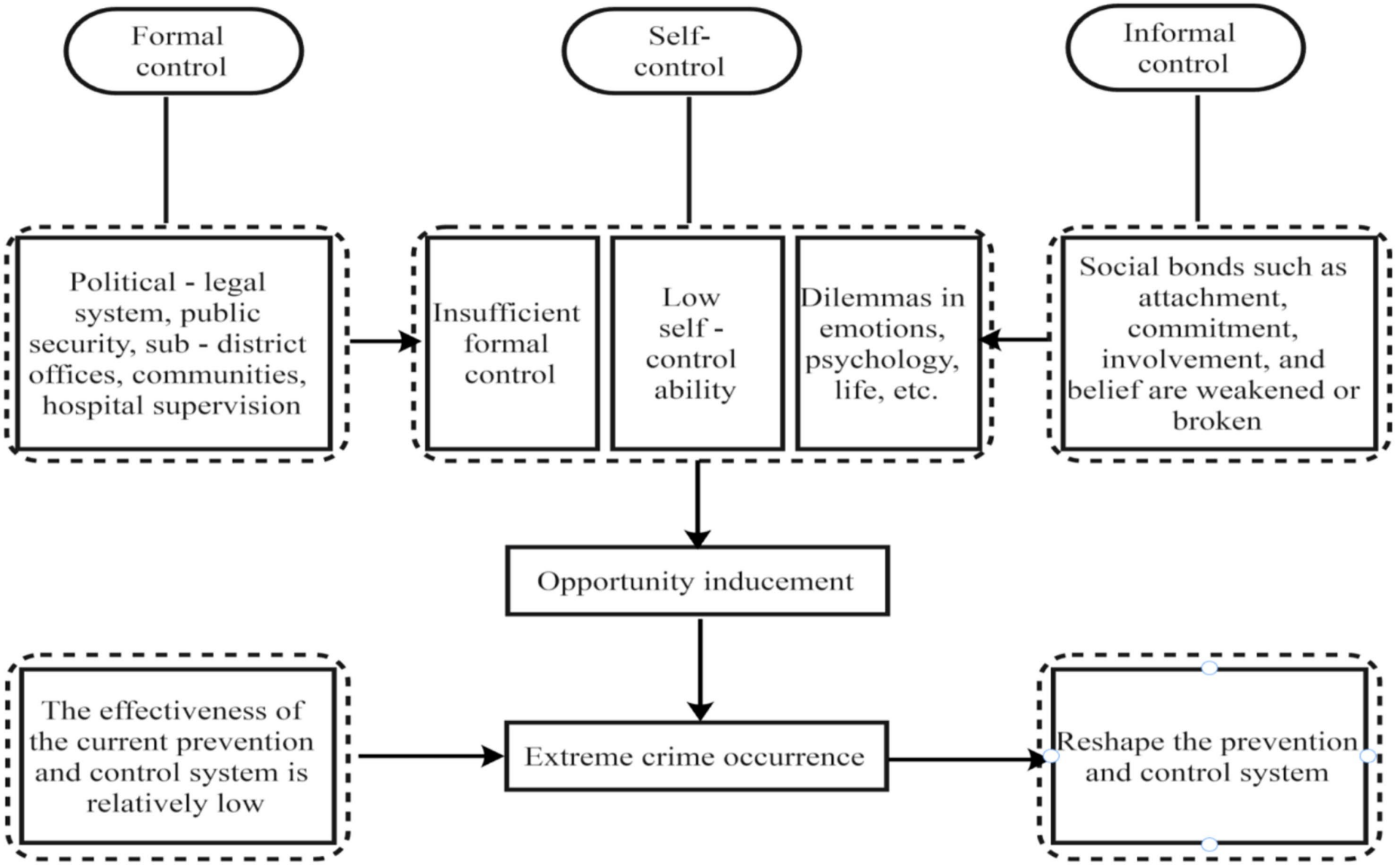
Theoretical framework.

The theory of social control is widely applied in criminological research, but it primarily focuses on issues such as fear of violent crime (Hwang and Hyun, 2022), juvenile delinquency (Prince, 2024), gun-related crimes (Cwick and Williams, 2024), animal-related crimes (Lynch and Genco, 2024), and community crimes (Azevedo et al., 2021), with a lack of research on individual extreme violent crimes. Meanwhile, research on individual extreme violent crimes in China mostly remains at the theoretical level, lacking empirical analysis. Based on the theory of social control, this study focuses on three types of social control: formal control, informal control, and self-control. As mentioned in the introduction, this study combines the Chinese context to establish an evaluation model for the prevention and control of individual extreme violent crimes based on the theoretical framework of social control, focusing on these three aspects (see Figure 1). First, formal control refers to state-established administrative departments such as the political and legal system, police, streets, communities, and psychiatric hospitals. Second, informal control mainly refers to individuals who influence the ethics and morality of criminal suspects through various social ties, encompassing the suspect’s parents, relatives, friends, teachers, employers (work), income, social customs, and so forth. Third, self-control includes whether the criminal suspect is anxious and irritable and whether they have bad habits such as alcohol addiction, gambling, and so on. This study will construct an evaluation model for the prevention and control of individual extreme violent crimes from these three dimensions.

### 2.2 Individual extreme crime assessment model

#### 2.2.1 Model establishment method

**Figure 2:**
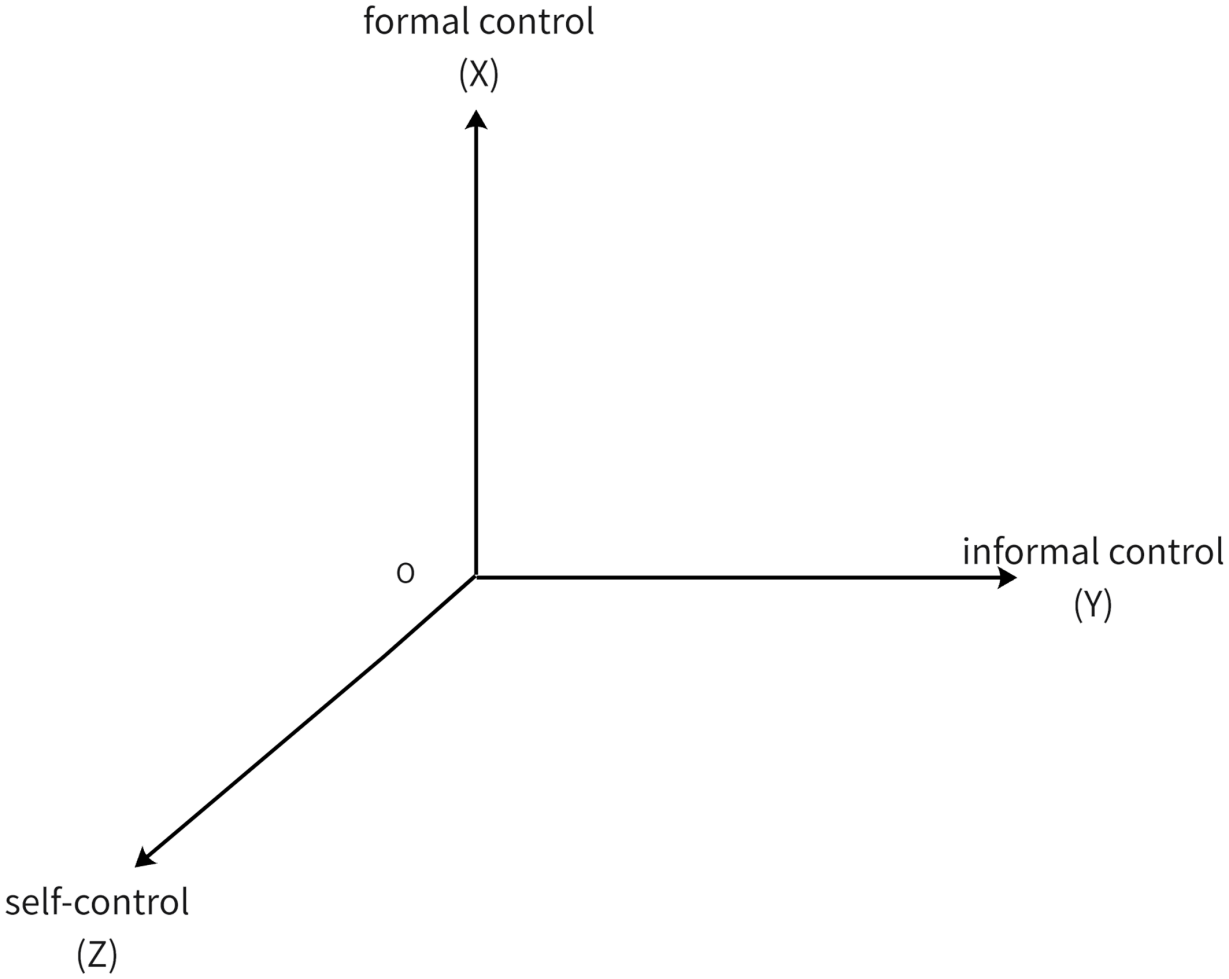
Rectangular coordinate system for prevention and control of individual extreme violent crimes.

**Figure 3:**
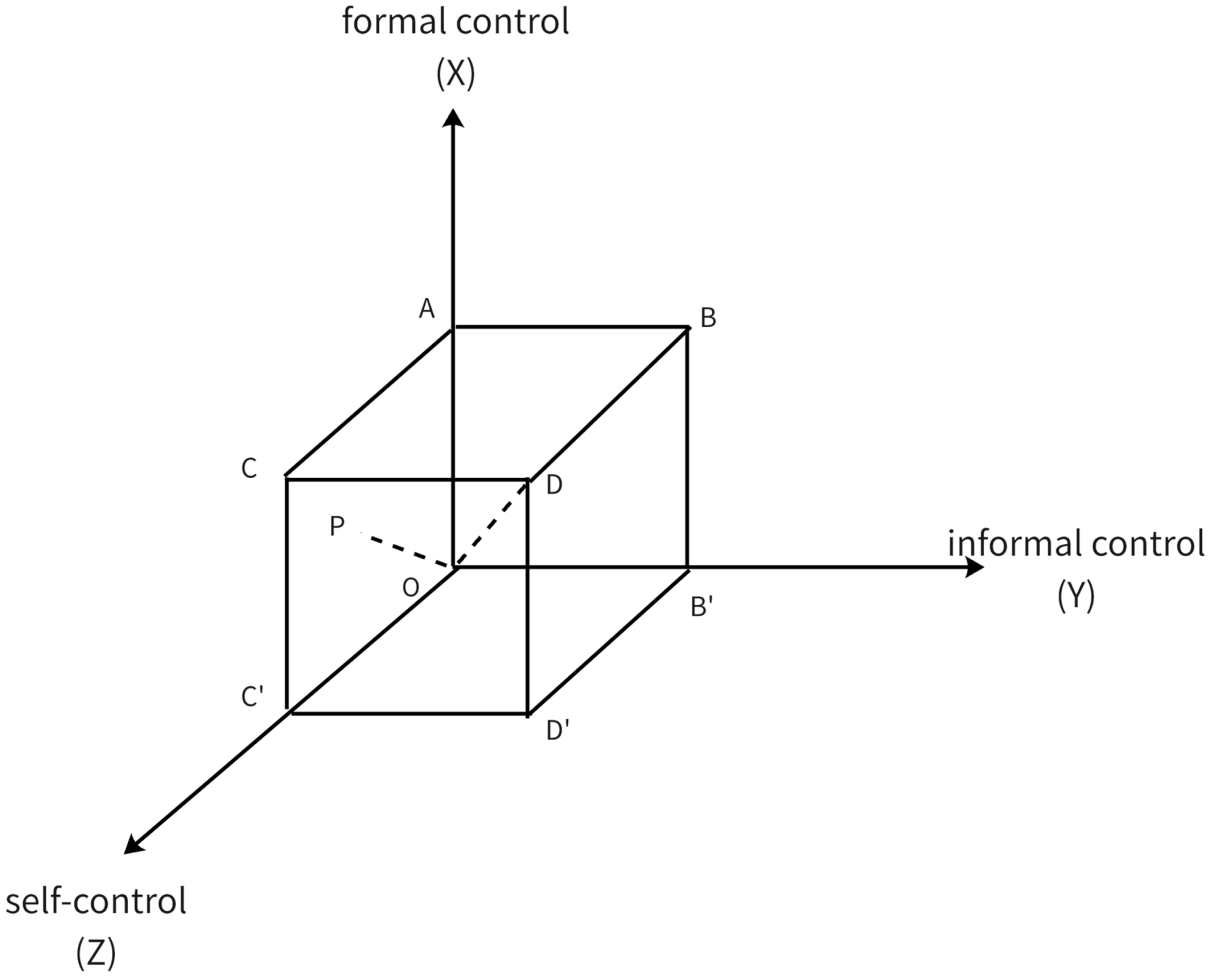
Assessment model for the prevention and control of individual extreme violent crimes.

In summary, crime control primarily encompasses formal control, informal control, and self-control. Based on this, the present study employs a spatial rectangular coordinate system (Wang, 2021) to construct an evaluation model for the prevention and control of individual extreme violent crimes, with formal control as the X-axis, informal control as the Y-axis, and self-control as the Z-axis (see Figure 2). In the three planes of ZOX, ZOY, and XOY, due to the absence of informal control, self-control, and formal control respectively, individual extreme violent crimes are highly likely to occur. Only within the space enclosed by the X, Y, and Z axes, where formal control, informal control, and self-control simultaneously exert control over crime, is the probability of crime occurrence relatively low. Based on this, a “crime control cube” is established within the spatial rectangular coordinate system, corresponding to the cubic region “ABCD-OB′C′D′” in Figure 3. The distance from any point within the coordinates of each point of the cube to the origin O represents the effectiveness of extreme crime prevention and control, denoted as PO. The magnitude of control effectiveness can be calculated using the following formula: $\mathrm{PO}=\sqrt{X^{2}+Y^{2}+Z^{2}}$ . From this, it can be seen that as the values of X, Y, and Z increase, the preventive and control capabilities of these three elements against crime also increase, and the likelihood of crime occurrence gradually decreases. As shown in Figure 3, point D on the diagonal of the cube has the greatest distance from the origin, at which point formal control, informal control, and individual self-control reach their maximum capabilities in preventing and controlling crime. At this point, individual extreme violent crimes are least likely to occur.

#### 2.2.2 Methods for quantifying coordinate values and rating levels

Formal control, informal control, and self-control are abstract concepts, and their specific measurements in a coordinate system need to be expressed and embodied through certain elements or data. Therefore, numerical values need to be assigned to these three types of control to calculate and compare the crime prevention and control capabilities at various points within the cube of the coordinate system. The Royal Canadian Mounted Police (RCMP) previously used the “Disruption Assessment Tool” (DAT) (Ratcliffe, 2016) to classify the core business, finance, and personnel characteristics of organized crime into six levels: high, medium, low, absent, not applicable, and unable to obtain or under investigation, to measure the effectiveness of disruption efforts against organized crime gangs (Ratcliffe et al., 2014). This system is designed primarily as an operational framework to systematically translate complex, qualitative fieldwork data into a quantifiable and comparable metric. Drawing inspiration from the DAT evaluation method, we rate the prevention and control effectiveness of formal control, informal control, and (note: “formal control” is repeated here, I assume it should be “self-control” for diversity) self-control. Excluding the two uncertain factors of “not applicable” and “unable to obtain or under investigation,” the prevention and control effectiveness of the three prevention and control entities is divided into four levels, with level 1 assigned a score of 0, level 2 assigned a score of 1, level 3 assigned a score of 2, and level 4 assigned a score of 3, as shown in Table 1. A higher numerical value represents a higher prevention and control effectiveness, which means a lower probability of the crime occurring. The range of prevention and control capability values is between 0 and $\sqrt{27}$ . Assuming an evaluation of the prevention and control capability value for individual extreme violent crime cases in a certain location, with the relevant factors of the three prevention and control entities assessed as level 2, level 3, and level 4 respectively, then the prevention and control effectiveness for this crime would be $\sqrt{14}$ .

**Table 1:** Assessment levels and score assignment for the prevention and control of individual extreme violent crimes.

Level	Score assignment	Formal control	Informal control	Self-control
High (level 4)	3	There are almost no regulatory defects	Almost no negative impact on crime	Almost never lose control of himself
Medium (level 3)	2	It has a greater regulatory role and fewer defects	It has a certain negative impact on crime, but it has little impact	Have strong self-control ability
Low (level 2)	1	There is some supervision, but there are major deficiencies	Have a greater negative impact on crime	Self-control, but impulsive
None (level 1)	0	It has no regulatory effect on crime	Promote or let the crime happen	Self-control ability is extremely low

In real life, formal control, informal control, and self-control all exert influences on individual extreme violent crimes through multiple aspects. Therefore, it is inappropriate to consider only one aspect; instead, a comprehensive assessment should be conducted. Consequently, for individual extreme violent crimes, several assessment factors are established under the three types of control. These factors are rated as follows: Level 0 for those with 0–25% of the factors met, Level 1 for 25–50%, Level 2 for 50–75%, and Level 3 for 75–100%. In the following text, relevant assessment factors will be elaborated regarding four cases of individual extreme violent crimes in China, to apply and test the model.

## 3 Research cases and research methods

The primary objective of this study was to conduct an in-depth, exploratory analysis to develop and initially test a new theoretical assessment model. The qualitative multi-case study method can capture the rich, contextual details of the suspects’ life histories and the intricate failures of the social control systems—details that a larger-scale study might miss. Although individual extreme violent crime cases have a significant social impact, the number of occurrences each year is relatively small, which limits the feasibility of large-scale quantitative analysis. Secondly, data acquisition is a significant challenge that requires professional credibility and a commitment of time, as it involves gaining the trust of law enforcement agencies and interviewees, and navigating complex administrative procedures to obtain official case files, including prosecution records and interrogation transcripts. In summary, the in-depth qualitative case study approach adopted by this research is highly necessary.

The cases in this study primarily originate from field investigations conducted between June 2021 and August 2022 into extreme crime incidents in four locations: SJ, NY, GS, and LP. The research team collected and organized data on four typical cases, with all perpetrators being male, aged between 28 and 50. Each incident involved a single perpetrator and resulted in serious injury or death to three or more people. The weapons used in the crimes were cars, sticks, knives, and gas, respectively. Two incidents occurred in indoor spaces, while the other two took place in outdoor public spaces, as shown in Table 2. Specifically, this study employed four methods: participant observation, focus group interviews, artifact analysis, and expert scoring.

**Table 2:** Brief introduction to four cases of individual extreme violent crimes.

Case number	Crime time	Crime site	Behavioral characteristics	The motive for the murder	Mode of crime	Crime scene	Casualties
1	20xx.7	SJ	X is a 41-43-year-old male with no stable occupation	Revenge on one’s ex-wife and society	Hit someone with a car	Public place	7–10 people were injured, four were critically ill, one was his ex-wife, and the other seven were unknown
2	20xx.4	NY	L, a 34-36-year-old male with a history of mental illness, is a farmer	Gambling habits, being kicked out of the “home” by his father-in-law’s family, and drinking alcohol caused a sudden mental illness that could not be controlled	Indiscriminate stick attack	Public place	3–5 people died, residents of the same village, no contradiction.
3	20xx.9	GS	H, a 28-30-year-old male, is an employee of an ordinary enterprise and has gambling habits	Borrowing and gambling, inability to repay, burglary	Stab with a knife	Indoor of residents’ houses	3–5 people died and one was seriously injured. All four people were from one family, including a 70-year-old man and a 2-year-old child
4	20xx.11	PL	F, 50–52 years old, male, unemployed	Economic disputes, the actor cannot repay the loan, and the victim has lived in the actor’s home for a long time, resulting in hatred	Block the gas outlet	Indoor of residents’ houses	4–6 people died, two old people and three young children

Firstly, field notes centered on participatory observation constitute a significant data source for this study (Roque et al., 2024). Institutional controls embodied in the provincial public security departments, political and legal committees, and police bureaus of various cities and counties, as well as physical and technical controls represented by communities and facilities, along with the family members, neighbors, and friends who constitute the main social networks of criminal suspects, are all objects of observation and recording in this study. Meanwhile, the interactive relationships among social controls of different dimensions and how they function in crime control are also key research and analysis focuses of the author in the fieldwork.

Secondly, for in-depth interviews, the study adopts a focus group approach (Zaharia and Zaharia, 2017). The interviews are conducted face-to-face, with each session lasting between half an hour to 2 h. The interviews focus on three main themes. Firstly, the basic information about the suspects, the criminal process, and the characteristics of the cases. Secondly, the respondents’ interactive experiences with institutional, physical, technological, and social controls in the four regions, the existence of any control loopholes, and the difficulties faced by the suspects. Thirdly, the life histories and personality traits of the suspects. Furthermore, to gain a deeper understanding of the life circumstances of the criminal suspects, the author also interviewed other relevant subjects associated with the suspects, such as their relatives, friends, neighbors, landlords, etc.

The present study employs the method of object analysis (Trausan-Matu and Slotta, 2021), which involves the recording and acquisition of official documents and related images. The official documents encompass policy papers and statistical data issued by government agencies at various levels, about social governance and social control. Furthermore, through conducting visual fieldwork, the author has photographed and documented relevant materials, generating a vast amount of image information to aid in the analysis and interpretation of data obtained from participant observations and interviews.

Fourth, expert evaluation and scoring (Delphi method) (Humphrey-Murto et al., 2020). This method involves objectively synthesizing the expert experience and subjective judgments of the research team to assign scores to various indicators of informal control and self-control, based on the collection and categorical analysis of various types of data. This scoring system is used to assess the magnitude of the impact of each factor and subsequently evaluate the effectiveness of prevention and control. The expert panel consists of five members, who use a relative importance scale of 1–5 points for scoring, representing: very low, low, moderate, high, and very high, respectively. Based on this scoring, informal control and self-control are rated separately. To ensure transparency and rigor, the expert panel consisted of five members selected based on specific criteria: an academic background in criminology or sociology, and over 5 years of professional experience in either criminal investigation, public security management, or grassroots social governance. The research team provided each expert with comprehensive, anonymized case summaries and a detailed scoring rubric. The experts conducted their scoring independently in the first round. Subsequently, a moderated discussion was held to allow experts to debate any significant discrepancies in their scores, ensuring a more robust and consolidated final judgment. The final scores presented in this study are the average ratings following this consensus-building process.

Finally, the effectiveness of prevention and control is assessed by integrating the ratings of the three types of control.

It should be clarified that any information regarding the suspects’ mental health status, such as a pre-existing history of mental illness, was not obtained through direct psychiatric evaluation by the research team. Instead, this information was sourced from official documents gathered during fieldwork, including prosecution records, interrogation transcripts, and interviews with family members who reported prior diagnoses or observable conditions.

## 4 Model application: the social control landscape of suspects and its efficacy

### 4.1 Formal control: institutional defects lead to blind spots in prevention and control

Since the Chinese Ministry of Public Security endorsed and vigorously promoted the slogan “All homicide cases must be solved” in 2004 (Liu, 2019). China’s public security situation has significantly improved, with a substantial decrease in the number of traditional homicide cases. Police specialties such as criminal investigation, technical investigation, cyber investigation, and image investigation have clear divisions of labor and cooperation, providing a robust institutional guarantee for case solving. However, reality indicates that while personal extreme violent crime cases are not difficult to solve, the problem lies in their prevention. Combining data obtained from field research, institutional factors affecting the prevention of personal extreme crimes are classified as formal control conditions. This study divides them into two aspects: the public security system and the local management system. By evaluating specific conditions in both aspects, the “positive impact” or “negative impact” of formal control is judged, totaling 11 items, as shown in Table 3.

**Table 3:** Formal control expert scoring.

Number	Condition	Have a great positive influence	Have a greater positive impact	No influence	The negative impact is greater	Have a great negative impact
1	Cause analysis of handling police affairs		4			
2	Failure to assess the risk level of police handling.					1
3	There are no guidelines for the prevention and control of personal extreme crimes.					1
4	The number of full-time community grid workers is insufficient.				2	
5	The data reported by community grid members is inaccurate.					1
6	The investigation of community contradictions and disputes is not timely.					1
7	There is no permanent linkage between politics and law, public security, women’s federations, and communities.					1
8	The data on politics and law, public security, women’s federations, and communities are not shared.				2	
9	Individual extreme crime police handling plan is complete.			3		
10	Police manpower, material resources, and equipment are fully equipped.			3		
11	Police officers are skilled in handling drills.			3		
Total (item)	11		1	3	2	5

Specifically, within the framework of the police system, personal extreme violent crimes can be categorized by their causes into emotional, economic, socially conflicted, workplace dispute-related, and psychiatric types, among others (Li et al., 2024). In the practice of prevention and control, the primary challenge faced lies in the strong concealment of these crimes, particularly those of emotional personal extreme violent nature. Unlike in Western countries (Green et al., 2024), in the Chinese cultural context, disputes between spouses are often perceived as “family matters” or “trivial issues,” which do not readily trigger the vigilance or intervention of police and community managers. This is similar to the situation in South Asian countries (De Silva et al., 2024). Despite the current adequate police staffing and equipment levels, as well as the frequent conduct of emergency drills, these measures primarily focus on post-incident response, with limited effectiveness in pre-incident prevention. The protection of personal privacy rights also poses a significant obstacle to the prevention and control of personal extreme violent crimes, making it difficult for police to timely, comprehensively, and effectively obtain early warning information about extreme crimes. The inadequacy of technical means, the absence of legal regulations, and the high costs required for comprehensive control jointly pose difficulties for police at the prevention stage. Secondly, improper handling methods are also important factors that affect the effectiveness of prevention and control. A significant proportion of personal extreme violent crimes are preceded by police reports. However, when handling these reports, police officers often respond by mediating ordinary disputes and resolving conflicts, adopting a “treating the symptoms rather than the root cause” approach that fails to fundamentally address the underlying conflicts. The insufficient assessment of conflicts and disputes by the police makes it difficult to accurately predict the risk of individual extreme violent crimes. Furthermore, unlike the United States, where the widespread availability of firearms is positively correlated with homicide rates and mass shootings (Stroebe et al., 2024), China has effectively reduced the occurrence of related violent incidents through strict control of firearms and restricted knives (Skiba, 2024). However, vehicles and other modes of transportation have increasingly become the primary tools used by criminals, a trend similar to changes in the modus operandi observed in terrorist attacks (Miller and Hayward, 2019), hate crimes, or mass murders (Mamuji, 2020). Moreover, the lack of effective coordination among multiple departments poses a major challenge in prevention and control efforts. The managers and data from departments such as politics and law, communities, and women’s federations have not been effectively integrated and shared, resulting in severe information isolation. In summary, the strong concealment of personal extreme violent crimes, deficiencies in the police response system, and the absence of a multi-department coordination mechanism collectively pose severe challenges to the current prevention system, significantly hindering the early detection and warning of personal extreme violent crimes. For instance, an interviewee named Cheng (male, 27 years old, a police officer in an eastern city of China) shared his experiences in handling police reports with the author, highlighting the concrete manifestations of the aforementioned issues in practical operations.

*“Before the incident, the victim reported the case to the police twice, once in X City and once in J City. During the incident in J, the victim called the police claiming that her ex-husband had threatened her and made physical gestures. When our policemen arrived at the scene, the emotions of both parties had already calmed down. Under the persuasion of the policemen, the suspect expressed that he would no longer harass the victim and that they would resolve their conflicts properly. His attitude was relatively sincere. The victim also accepted the suspect’s apology at that time. “After handling the situation, the policeman completed the necessary records and concluded the case.”* (Case SJ: Local police at the scene of the incident).

At the local administrative level, the collection and screening of risk information constitute an important means of preventing personal extreme violent crimes. In China, grid managers have increasingly become an indispensable force in grassroots social governance. They undertake multiple tasks including publicity, information collection, conflict resolution, and assistance in management (Chen et al., 2024). Currently, there are four main issues in their work: Firstly, community grid managers are not fixed in personnel and have high mobility, resulting in inconsistent data reporting. Secondly, a large amount of reported data is repeated and invalid, often submitted solely to meet inspection requirements or complete tasks. Thirdly, community data is not effectively correlated with various police situations. Fourthly, in vast rural areas or urban–rural fringe zones, there are no grid managers at all. Many of the individuals involved in cases are migrants who reside long-term in resettlement communities within the incident cities due to cheap rent. These communities are characterized by a diverse and complex population and chaotic, lagging property management. Interviewee Wang (male, 36 years old, a street-level cadre from a western city in China) shared with the author the current work situation of grassroots community grid managers.

*“The monthly salary for a full-time grid manager in our area is only 1,500 yuan. This amount of money can only barely sustain one’s living expenses nowadays. Therefore, many people are unwilling to take up this job, and even if they do, they will not stay for long. It lacks appeal, and there is no dedicated personnel to handle this task. Consequently, much information is incompletely collected or simply submitted arbitrarily. There are numerous forms to fill out at the grassroots level, making the work quite cumbersome. “As for how these data are utilized after being reported, we are unaware.”* (Case PL: Deputy Director of the Sub-district Office where the incident occurred).

The statements made by Officer Cheng and Director Wang, as well as the experiences reflected by the relevant personnel, all indicate the existence of certain obstacles within the current prevention system, which hinder the resolution of issues related to both parties involved. In fact, interviewees from other cases in this study have also shared similar viewpoints. The analysis of the above-mentioned systems and cases reveals that while the grassroots management system, represented by police officers and community grid managers, deals with a multitude of “major and minor issues,” it fails to promptly and effectively identify risk points associated with some potential perpetrators of personal extreme crimes. On the one hand, the current risk warning system is inadequate, and police officers are unable to collect detailed information on non-key personnel in their daily work. Community grid managers face significant deficiencies in staffing, data collection, coordination, and utilization (Jia, 2022), which enhances the “suddenness” of personal extreme violent crimes. On the other hand, when handling potential personal extreme crimes, police officers still treat the involved parties as if they were involved in routine disputes during the process of responding to calls and handling incidents, without conducting risk assessments. This further prompts them to seek alternative paths for resolving issues.

From the comprehensive scoring by experts, the average score for formal control is only 2, thus rating the effectiveness of formal control as Level 0, indicating that negative impacts cannot effectively prevent the occurrence of personal extreme violent crime cases. Among the 11 conditions, there are 3 with no impact, mainly referring to police response work after the incident, including “complete response plans for personal extreme violent crime alerts,” “adequate police manpower, material resources, and equipment,” and “proficient police response drills and disposal.” There are 0 conditions with a significant positive impact and 1 with a relatively large positive impact, referring to “analysis of the causes of police responses.” There are 2 conditions with a relatively large negative impact, namely “insufficient full-time community grid managers” and “lack of data sharing among political and legal affairs departments, public security departments, women’s federations, and communities.” There are as many as 5 conditions with a large negative impact, including “failure to conduct risk level assessments for police responses,” “lack of a police prevention and control guide for personal extreme crimes,” “inaccurate data reporting by community grid managers,” “untimely investigation and mediation of community conflicts and disputes,” and “lack of a permanent linkage mechanism among political and legal affairs departments, public security departments, women’s federations, and communities.”

It is crucial to note that the challenges in formal control do not stem from a legal vacuum. China enacted the “Anti-Domestic Violence Law of the People’s Republic of China” in 2016, providing a clear legal framework for intervention. This law empowers police to issue official warnings and allows victims to apply for personal safety protection orders from the courts. However, as the cases in this study suggest, a significant gap exists between the legal provisions and their grassroots-level implementation. Influenced by the cultural concept of “persuading to reconcile, not to separate,” if the woman is unwilling to go to the hospital for an injury assessment or has no visible injuries, coupled with the lack of professional training for frontline officers in risk assessment for individual extreme violent crimes, they often prioritize mediation over legal intervention. At the same time, the female victims may have weak legal awareness or find the process troublesome; when the police intervene, perpetrators often adopt a very sincere attitude, apologizing and admitting fault, leading the female victims in the cases to choose reconciliation under these circumstances. This highlights that the failure of formal control is not due to the absence of law, but a misalignment in practice, where institutional inertia and cultural norms impede the effective enforcement of the law.

### 4.2 Informal control: adding fuel to the fire and pushing them into a dilemma

Based on the analysis of data obtained through fieldwork, family and social factors that may influence crime are categorized as informal control conditions. The assessment of whether a particular family or social factor affects a specific type of crime is complex. This section determines “positive impact” or “negative impact” by measuring whether the condition has a clear and direct influence on personal extreme violent crime, as shown in Table 4. Among the 15 conditions, there are 3 with no impact, 9 with relatively large negative impacts, and 3 with significant negative impacts. “No criminal record among immediate family members,” “not influenced by criminal-themed movies or TV shows,” and “living in a harmonious community” all have no impact on extreme crimes. In the four cases investigated, the immediate family members of the suspects had no criminal records. Their parents were mostly honest and law-abiding farmers, and the community environments in which they grew up had no significant criminal cases. Unlike Reena’s view that violent movies are related to aggressive behavior (Shaktawat), the suspects had watched some martial arts or gangster movies, but these had little explicit or potential impact on them and were only used for entertainment. It is noteworthy that most of the suspects were migrants who entered society after completing middle or high school, meaning they left their hometowns or communities before adulthood and entered an unfamiliar society, where the original acquaintance-based social norm restraint mechanism was ineffective for them (Chen and Xu, 2025). Tang (male, 58 years old, from a rural area in eastern China) briefly introduced the birthplace of the suspects:

**Table 4:** Expert ratings of informal control.

Number	Condition	Have a great positive influence	Have a greater positive impact	No influence	The negative impact is greater	Have a great negative impact
1	Immediate family members have no criminal record.			3		
2	No fixed source of income				2	
3	Have no stable job				2	
4	Have not received a good education				2	
5	Not influenced by criminal film and television.			3		
6	Living in a harmonious community.			3		
7	Couples’ emotional breakdown/infidelity					1
8	Family persuasion				2	
9	Care for relatives and friends				2	
10	Have been discriminated against/abused					1
11	Family intergenerational relations are not harmonious.				2	
12	Media publicity on the rule of law is not in place.				2	
13	Traditional bad ideas are strong				2	
14	Have a major economic dispute					1
15	Less contact with relatives and friends				2	
Total (item)	15			3	9	3

*“Q seemed to have left home to seek his fortune after dropping out of junior high school in his second year. He first went to F, then to S and Z, and has been in X for the past few years, rarely returning home except for once during the Chinese New Year. He has changed a lot since he left and is different from those of us who have remained in the countryside. His parents are honest people who have always farmed at home and have never been involved in any misdeeds.”* (Neighbor from the suspect’s hometown in the SJ case).

Negative impacts can be categorized into three major groups. The first group involves fragile attachment bonds, which encompass the following four aspects: First, the negative effects of family persuasion. As the most crucial social institution, the family serves as a place for human growth, evolution, healing, and trauma recovery, playing a pivotal role in personality formation and socialization. The quality of family relationships influences interactions among family members (Javdan et al., 2023). However, family persuasion can sometimes exert negative effects. For instance, in emotional disputes, both parties’ relatives, particularly parents, tend to take their children’s side and vigorously belittle the other party, which instead intensifies conflicts between them. Second, the absence of care from relatives and friends. This is most evident in cases where the perpetrator of criminal behavior is a mental patient. Family/relative care is vital for mental patients. However, due to a lack of professional knowledge or a weak sense of responsibility, they may not provide meticulous care to the patient and may even resort to violent measures, further exacerbating the patient’s condition. Third, disharmonious intergenerational relationships within the family. Our research revealed that overly dominant parents who employ corporal punishment and verbal abuse in educating their children, as mentioned by Johnson as “patriarchal terrorism” (Johnson, 2017), often lead to the emergence of malformed personalities and character deficiencies in their children. Under the influence of other factors, these children may engage in extreme behaviors to retaliate against their family or society, which is what Moreno refers to as the “intergenerational transfer of violence” (Moreno Martín, 1999).

Fourth, infrequent interactions with relatives and friends. Interactions with relatives and friends also influence the behavior of suspects. If an individual has many relatives and friends and interacts with them frequently, they are generally less likely to become self-isolated. However, if the social ties of interaction are broken, the individual’s behavior or mental health may become problematic, which aligns with the research of Bellair (1997) and Carneiro et al. (2005), who found that strengthening an individual’s ties with their family and community reduces the probability of crime occurrence.

The second category is the disruption of investment, primarily referring to “having no fixed source of income,” “lacking stable employment,” and “not receiving a good education.” These individuals do not devote their primary time and energy to work or study. Coupled with the impact of the pandemic, many industries have been adversely affected, leading to a narrowing of employment opportunities. Consequently, the livelihoods of some groups have been impacted, survival pressures have increased, and social dissatisfaction has risen. Under the stimulation of situations such as a partner’s “infidelity involving money” or “online loan deadlines,” some individuals may be unable to bear the pressure and commit extreme acts. This aligns with the views of ROBERT and Julie, who argue that there is an inverse relationship between employment and crime and that time, income, and subjective investment in work are strongly correlated with criminal behavior (Apel and Horney, 2017).

The third category is the distortion of beliefs, which encompasses “inadequate media promotion of the rule of law” and “strong traditional negative concepts.” In some regions, traditional concepts remain deeply ingrained, such as the belief that “a blood feud against a father’s killer or a husband’s rival for his wife is unforgivable,” reflecting a strong male chauvinism. If a wife is unfaithful and this becomes known to others, the husband is often looked down upon, leading to a severe blow to his self-esteem. Coupled with inadequate media promotion of the rule of law and a weak sense of legal awareness, extreme ideas can dominate when stimulated. This is inconsistent with the research views of Barton and Jennifer, who argue that morality plays a significant moderating role in the pathway between partner conflict and partner violence. That is, individuals with weak moral values and frequent partner conflicts are more likely to engage in partner violence than those with strong moral values and frequent conflicts with their partners (Barton-Crosby, 2018). However, in the cases studied, it was precisely the criminals who possessed extremely strong traditional moral beliefs and could not tolerate their wives’ betrayal, leading to the motivation to commit extreme crimes.

*“After we disagreed, her parents blamed me entirely and advised their daughter to leave me. I originally worked in the microfinance industry, but it was later deemed illegal, resulting in unstable income. Now that I’m no longer in that field, I have no other significant sources of income. The money from selling our house was also taken by W (the victim). I had planned to learn to cook crayfish as a chef, but due to the pandemic, I started driving for DiDi for a few days instead. The income was not only low but also unstable. The fact that she cheated on me is something I, as a man, cannot tolerate, so I wanted to make her suffer moreis…”* (SJ Case: Suspect Q).

Three factors with significant negative impacts are: “marital discord/infidelity,” “experiencing discrimination/abuse,” and “having significant economic disputes.” These represent direct ruptures in social ties. In cases of marital discord/infidelity, most often involving female infidelity, one scenario is when the female party leaves home with money and children, and the male party, after being rejected in his attempts to reconcile, faces immense pressure in terms of economic rational costs, emotional cognition, and social reputation. Under situational stimulation, extreme behaviors may occur. Another scenario is when the male partner of the female infidelity seeks revenge. After the female party cheats, she becomes entangled in a triangular dispute and is abandoned after investing emotions, facing public condemnation, and experiencing strained family relationships. Having lost in the “competition” with the other male, she often develops extreme thoughts after arguing with him. This aligns with the research of Zhao Shuhong, who argues that the rapid increase in intimate partner homicide cases in China is mainly due to one party in the marriage maintaining a close relationship with someone outside the marriage. Moreover, this phenomenon is synchronized with the social and family changes brought about by China’s rapid modernization and urbanization (Zhao, 2022). “Experiencing discrimination/abuse” is more prevalent among “married sons-in-law who move into their wife’s family” (a phenomenon where the male, due to his lower economic status, marries into the female’s family). Such individuals face discrimination and even abuse both within the family and in society. In some places, the children take the mother’s surname, and in extreme cases, the married son-in-law is not even allowed to eat at the table with the family. This is similar to the research of Zhao Bo, who believes that oppression, discrimination, and the cessation of economic dependence in uxorilocal marriages are important factors triggering intimate partner femicide (IPF) (Zhang, 2024). Long-term repression can stimulate extreme behaviors among married sons-in-law. In addition to causing harm to women, the victims in extreme cases often expand to include the woman’s children, parents, close associates, or even innocent individuals. Wu (male, 47 years old, from a rural area in western China), who is familiar with the suspect’s family, was interviewed regarding his understanding of the situation.

*“The male individual hailed from a relatively impoverished family and became a married son-in-law in his wife’s family a few years ago. Subsequently, due to gambling, his marital relationship broke down, leading to his expulsion by his wife’s family. He suffered significant blows both in terms of income and dignity. Upon returning home, he frequently indulged in alcohol abuse and became increasingly negative. Over time, this gradually led to mental health issues.”* (NY Case: Acquaintance of the Suspect).

“Significant economic disputes” often arise from lending issues between parties, where borrowed funds remain unpaid for an extended period, coupled with a lack of legal awareness, leading to the escalation of conflicts and ultimately the occurrence of extreme cases. Wang (male, 38 years old, from a city in western China) is a township cadre at the location of the incident. He shared with the author the sequence of events leading to the incident:

*“The two families are relatives. The suspect borrowed over 200,000 yuan for business purposes, but due to poor business performance, failed to repay the loan upon maturity. According to the agreement signed by both parties at that time, the house was pledged as collateral. The victim, along with their grandchildren, directly moved into the suspect’s home, leading to the escalation of conflicts between the two parties. The suspect then blocked the exhaust pipe, resulting in excessive indoor gas levels and multiple fatalities.”* (GS Case: Township Cadre).

Based on the comprehensive scoring by experts, the average score for informal control is only 2 points. Therefore, the effectiveness of informal control is rated as Level 0, indicating that negative impacts facilitated or allowed the occurrence of extreme criminal cases.

### 4.3 Self - control: multi-dimensional factors lead to insufficient self-control ability

The author not only focuses on how formal institutional control and external social support influence the conflicts and disputes between the parties involved in a case but also emphasizes individuals’ proactive strategies in addressing obstacles, with self-control ability determining the choice of strategies. Self-control focuses on the criminal suspect’s personality and habits, with its setting conditions detailed in Table 5. One factor that has no impact is “no prior criminal record,” as all criminal suspects in the four cases investigated did not have a prior criminal record. Two factors with significant negative impacts are “irritability/domestic violence” and “inferiority complex/narrow-mindedness.” An important manifestation of low self-control ability in criminal suspects is their inability to effectively manage their emotions, leading to impulsiveness and domestic violence. Prolonged domestic violence is one of the significant reasons for women to divorce or have affairs. Inferiority complex and narrow-mindedness prevent individuals from correctly handling situations and escaping from a “self-imposed loop.” During interrogation, Ding (male, 42 years old, from an eastern city in China) and Peng (male, 36 years old, a native of Northeast China living in a western city) respectively reflected on their interaction patterns with their ex-wives/lovers.

**Table 5:** Expert ratings of self - control.

Number	Condition	Have a great positive influence	Have a greater positive impact	No influence	The negative impact is greater	Have a great negative impact
1	Heavy drinking					1
2	(Internet) Gambling					1
3	Irritability/domestic violence				2	
4	No criminal record			2		
5	Inferiority/narrow-mindedness				2	
Total (item)	5			1	2	2

*“We met through online chatting while we were both working as migrant workers. W has a higher educational background than me. She is well-mannered and has a good personality, and it is my fortune to have married her. She treats everyone in the family, both old and young, with kindness. It is I who am at fault. In my daily life, I enjoy having a few drinks, and when we argue, I tend to get emotional easily. I have hit ZJ before, and on some occasions, I was quite harsh. Especially after the issues of selling the house and taking out small loans arose, I felt even more upset and frustrated. “And I hit her more frequently.”* (SJ Case: Suspect Q).

*“Some people say I was the third party, but I disagree. At the time I was dating Z, she had already divorced. There were reasons related to why she reconciled with her ex-husband later on. Possibly, my possessiveness was too strong, and I had physically assaulted her, which was one of the reasons. However, I believe the bigger reason was her attachment to her two children. Therefore, she betrayed me, and I have been unable to move on. I am psychologically distressed.”* (LP Case: Suspect PZF).

The cases of Ding and Peng, coupled with the author’s fieldwork research, indicate that in romantic relationships, violence serves as a “modulator” of emotional life, not only causing significant physical and psychological harm to the victims but also having a reciprocal impact on the perpetrators themselves. That is to say, the violent behavior of perpetrators, on the one hand, creates emotional crises between the genders, fosters feelings of hatred, and deals a heavy blow to their inner selves. Rather than resolving issues, it often leads to “unintended consequences,” trapping individuals in a vicious cycle of “minor issues - violence - major issues - severe violence.” On the other hand, violent behavior also weakens the emotional and social support of perpetrators, particularly support from the victim’s family and friends. The combined impact of these two aspects can lead perpetrators to an “emotional dead end.”

There are also two factors with significant negative impacts: “excessive alcohol consumption” and “(online) gambling.” Excessive alcohol consumption can stimulate the nervous system to provoke extreme behavior, and chronic alcohol abuse can lead to various physical and mental illnesses (Harris, 2024). This may be related to accumulated work or life pressures and a lack of social support (Catalá-Miñana et al., 2017). Meanwhile, it often serves as a “trigger” for criminal behavior (Freestone et al., 2017). Michael et al. also believe that stricter control over alcohol consumption can reduce extremely violent behavior between intimate partners (Gottfredson and Nielsen, 2024). Gambling is also a vice, especially in recent years with online gambling leading to severe debt (Wijaya and Ananda, 2024). Gambling itself is an illegal act, often too embarrassing to discuss, and borrowing money through online platforms or other means to continue gambling creates a vicious cycle. Until one becomes unable to repay debts and cannot borrow anymore, leading to the idea of obtaining money through extreme means (Oksanen et al., 2018). Officer Mao (male, 38 years old, from a city in western China) briefed the author on the general process of the case:

*“X’s family background is relatively good. His father is an ‘unlicensed lawyer’ who helps people with legal disputes, earning around 300,000 yuan per year, which is considered high income in our area. The suspect’s house and car were fully paid for by his father. However, his father exercises strict control over him, and the suspect has no job. During COVID-19, he stayed at home, and in the year before the incident, he became involved in online gambling. He was deceived by a fraudulent gambling APP and lost 130,000 yuan. The money he used for gambling came from seven online lending platforms. On the day of the incident, unable to repay the loans, he conceived the idea of murdering someone to rob their wealth. “Ultimately, this led to three deaths and one injury.”* (GS Case: Case Handling Personnel).

As mentioned above, deficiencies in formal control and the weakening of informal control pose specific challenges for suspects in dealing with problems. Concurrently, personality issues and bad habits exert negative impacts on suspects’ daily lives, rendering them unable to cope normally when encountering significant changes or conflicts, and facing cognitive and behavioral barriers. In the face of adversity, neither suspects nor victims completely passively accept their situations; instead, they attempt to reconstruct their relationship through a series of proactive strategic actions. As evidenced by past cases, constrained by various conditions, suspects accumulate conflicts over time and ultimately choose the path of personal extreme violence under sudden circumstances or external stimuli. It should be noted that in the current rapidly developing society, individuals in society, especially members of the lower-middle class, inevitably face constraints and limitations imposed by a series of structural factors at macro, meso, and micro levels. Among these, differences in sociodemographic attributes and self-control abilities at the individual level can lead to variations in the choice of problem-solving pathways.

In the context of extreme crimes, self-control does not play an effective role. The average score is 1.6. Therefore, it is rated as Level 0. Low self-control ability is unable to prevent the occurrence of extreme crimes.

### 4.4 Comprehensive comparison: evaluation of the prevention and control efficacy of individual extreme crimes

Based on the ratings of the above three elements, we assess the preventive effectiveness against individual extreme crimes and evaluate the effectiveness of the current social control system. The preventive effectiveness against individual extreme crimes is set as point P, with coordinates (x, y, z) based on the levels of formal control, informal control, and self-control. The distance between point P and the origin O is calculated accordingly. The effectiveness of the three dimensions of social control is divided into four levels, represented by numbers 0–3, where a higher number indicates a higher level, which means better preventive effectiveness and a lower probability of individual extreme crimes occurring. The ideal state of preventive effectiveness is (3,3,3). If the prevention and control factors of a certain area are rated as Level 2, Level 3, and Level 4, then its coordinates are (1,2,3), and the effectiveness of preventing individual extreme crimes in that area would be $\mathrm{PO}=\sqrt{1^{2}+2^{2}+3^{2}}=\sqrt{14}$ . In this study, the levels of formal control, informal control, and self-control mentioned above are all Level 0, $\mathrm{PO}=\sqrt{0^{2}+0^{2}+0^{2}}=0$ , which means that the prevention systems in the four areas are ineffective. Without intervention and change, the possibility of individual extreme crimes occurring in the short term remains high. Overall, the current prevention and control of individual extreme violent crimes still mainly focuses on formal control with the police as the main body. However, formal control emphasizes post-incident response, with insufficient pre-incident prevention, and informal control and self-control hardly play a role.

Based on the number of individual extreme crimes in four regions in 2023, although there are variations compared to 2021, 2020, 2019, and 2018, the fluctuations are not significant, and there is no apparent downward trend. Nationwide, multiple extreme homicide cases involving “one killer, multiple victims” have occurred in the first quarter of 2024. Examples include the “1.31” case in Qujing (6 deaths) and the “5.7” case in Zhaotong (2 deaths and 23 injuries). In November 2024, there were consecutive extreme cases such as the “11.11” case in Zhuhai (35 deaths) and the “11.16” case in Wuxi (8 deaths). This indirectly supports the hypothesis of this study that, currently, effective institutional systems for preventing individual extreme crimes have not been established nationwide.

## 5 Discussion

This study’s findings offer a granular view of how social control failures contribute to individual extreme violent crimes. Beyond the specific ratings, a deeper discussion is warranted on the interplay of control mechanisms, the role of gender, and the unique ways in which social control theory manifests in the contemporary Chinese context and its connection with psychology.

### 5.1 The interplay of control failures

A critical insight from analysis is that the failures of formal, informal, and self-control are not independent but are dynamically interconnected. The quantitative model, for simplicity, treats them as orthogonal axes, but the qualitative evidence reveals a cascading effect. A breakdown in informal control—such as job loss or marital infidelity (weakened commitment and attachment)—often acts as the initial trigger. This breakdown directly erodes an individual’s self-control, leading to emotional dysregulation, substance abuse, and impulsive behavior. For instance, in the SJ case, the suspect’s marital breakdown led to a state of emotional distress that made his violent impulses difficult to manage. This weakened self-control, in turn, renders formal control interventions, like police mediation, insufficient. Officers responding to a family matter may fail to perceive the underlying self-regulatory collapse, leading them to underestimate the risk of severe escalation. This demonstrates a vicious cycle where failures in one domain amplify failures in others, leading to a total collapse of the prevention system.

### 5.2 Gender, masculinity, and social control

The fact that all perpetrators in cases are male is a finding that requires specific attention. This empirical reality suggests that the breakdown of social control is deeply gendered. In the Chinese context, traditional notions of masculinity are often tied to a man’s ability to be a successful economic provider and the undisputed head of his household. The failures of informal control identified in this study—such as unemployment, significant debt, or a wife’s infidelity—represent direct threats to this masculine identity. For individuals with low self-control, such threats can be perceived as an intolerable loss of honor and status, leading to extreme violence as a means to reassert control and save face. The violence is thus not merely an impulsive act but a distorted performance of masculinity in the face of perceived social and personal failure. Future research should more deeply explore the intersection of gender roles and social control theory in explaining IEVCs.

### 5.3 Social control theory in the Chinese context

This study also highlights the need to adapt social control theory to China’s unique socio-political landscape. The concept of weakened informal social bonds, for example, is powerfully illustrated by the erosion of the traditional acquaintance society. Decades of rapid urbanization and internal migration have moved millions from tight-knit rural communities to anonymous urban environments, severing the deep, multi-generational social ties that historically enforced social norms. Furthermore, the failures of formal control are intrinsically linked to the dynamics of China’s grassroots governance, particularly the grid management system. While designed to extend state oversight, this system often struggles with high personnel turnover and inaccurate data collection, especially in marginalized communities, proving ineffective at recreating the organic social control of the past. This analysis shows that a simple application of Western theory is insufficient without a deep contextualization in China’s ongoing social transformation.

### 5.4 Expanding the framework: integrating psychological variables

It is important to acknowledge that the analytical framework of this study is primarily rooted in the sociological tradition of social control theory. Within this framework, “self-control” serves as the main individual-level psychological component. However, a more comprehensive understanding of extreme violence could be achieved by integrating a wider range of psychological variables. Factors such as a perpetrator’s trauma history, their specific coping strategies for dealing with life stressors, and potential pathological personality traits are undoubtedly critical in explaining the pathway from grievance to extreme violence. For instance, an individual’s inability to cope with the shame of marital breakdown or economic failure, possibly rooted in past trauma, could be a more direct predictor of their low self-control and subsequent aggression. Integrating these elements would transition the current framework into a more powerful socio-psychological model. While a full integration requires a distinct interdisciplinary methodology beyond the scope of this paper, we strongly advocate for future research to bridge this gap. Combining the sociological insights of our social control model with established psychological frameworks represents a vital next step toward a more holistic understanding and prevention of these tragic events.

## 6 Conclusion

This study, grounded in fieldwork on individual extreme violent cases, focuses on exploring institutional control, the daily difficulties of those involved in the cases, and suspects’ self-control abilities from three dimensions: formal control, informal control, and self-control. By analyzing real-world cases, it assesses the current situation of low effectiveness in extreme crime prevention and control systems at the locations of incidents. On this basis, it also details the respective influencing factors of these three dimensions: In terms of formal social control, the five factors with the greatest negative impacts are “failure to conduct risk level assessments for police responses,” “lack of police prevention and control guidelines for individual extreme crimes,” “inaccurate data reporting by community grid managers,” “untimely identification and resolution of community conflicts and disputes,” and “lack of permanent inter-agency collaboration among political and legal affairs departments, public security organs, women’s federations, and communities.” In terms of informal social control, the three factors that can directly lead to the breakdown of social ties are “marital discord or infidelity,” “discrimination or abuse experienced,” and “significant economic disputes.” In terms of self-control, alcohol abuse and gambling are the most influential bad habits. Significant deficiencies exist in all three control dimensions, making individuals susceptible to forming extreme personal crimes when catalyzed by specific situational opportunities and incentives.

In the process of preventing and controlling individual extreme violent crimes, the following three aspects can be strengthened: (1) Reforming police response protocols: To counter the inadequacy of simple mediation in high-risk situations, police departments should develop and implement a mandatory, evidence-based risk assessment checklist for officers responding to domestic disputes, threats, and harassment calls. This tool would help frontline officers identify warning signs of potential escalation and trigger more formal interventions as prescribed by law. (2) Enhancing grassroots governance efficacy: To overcome the information silos and data inaccuracies of the current grid management system, a formalized inter-agency data-sharing and early-warning protocol should be established. This system would link data from community grid managers, police records, social service agencies, and women’s federations to create a holistic view of at-risk individuals and families, enabling proactive rather than reactive interventions. (3) Establishing community-based crisis intervention: To address the ruptures in informal control and failures in self-control, government and civil society should pilot community-level crisis intervention programs. These services would offer immediate, accessible support—such as emergency psychological counseling, temporary housing, distribution of IEVC response guidelines, and legal aid—for individuals facing acute life stressors identified in our study as triggers for violence, including severe economic disputes, unemployment, or acute marital breakdowns.

Based on this, the main contributions of this study are embodied in the following two aspects. Firstly, at the theoretical level, by focusing on “formal control,” “informal control,” and “self-control,” this study expands and enriches the strength, depth, and breadth of social control as an important theoretical analysis framework in the field of criminology. Firstly, most existing studies focus on the regulation and restraint of suspects by social control within the macro-institutional context. In contrast, this study emphasizes the pre-crime stage, deeply analyzing and exploring how social control shapes and influences individual behavior before the crime commission. Secondly, studies within the framework of social control typically emphasize the institutional control dimension within geopolitical and socio-economic frameworks. This study also explores the various difficulties faced by suspects in terms of emotions, rational survival costs, and psychology. Furthermore, this study also pays attention to the role of individual self-control abilities in extreme crimes. Secondly, at the empirical level, existing studies on individual extreme crimes mostly adopt a macro perspective, focusing on topics such as the characteristics, causes, and prevention of extreme crimes, with relatively little attention paid to the individual level of suspects. Therefore, this study focuses on exploring the prevention and control of extreme crimes through micro-level social control from a daily perspective, thus presenting a more detailed and comprehensive multi-dimensional view of extreme crimes, especially showcasing the life courses of the relevant individuals. This study provides important empirical insights and references for the further improvement and development of the national social governance system. Individual extreme crime poses a common challenge to global social governance, and its causes and prevention require the integration of regional social contexts with theoretical deepening.

The author acknowledge three limitations in this study that offer avenues for future research. First, as a qualitative study based on four cases from specific regions, the findings are not statistically generalizable to all of China. The primary goal of this research was exploratory—to build and test a novel assessment model through in-depth analysis rather than to make broad, generalizable claims. Future research should employ a larger, nationally representative sample to validate the model and explore the urban/rural and regional variations that this study could not capture. Second, the assessment model and its scoring system, while useful as a heuristic tool for structuring analysis, have inherent subjectivity. The quantification of qualitative concepts remains an approximation. A crucial next step for this research agenda is to develop a survey instrument based on our qualitative findings. This would allow the model’s complex relationships to be tested more rigorously using advanced quantitative techniques like Structural Equation Modeling (SEM), which could assess the dynamic interactions and relative weights of the three control dimensions. Third, This study’s deliberate focus on the under-researched Chinese context is intended to lay the necessary foundation for future, meaningful cross-cultural comparisons. A key future direction is to apply and test this model in other cultural contexts (e.g., Japan, South Korea, and Western nations). Such international collaboration would help distinguish the universal drivers of social control failure from culturally-specific factors, thereby enhancing the model’s external validity and global relevance.

## Data Availability

The original contributions presented in the study are included in the article/supplementary material, further inquiries can be directed to the corresponding author.
